# School functioning of children with perinatal HIV-infection in high-income countries: A systematic review

**DOI:** 10.1371/journal.pone.0252746

**Published:** 2021-06-04

**Authors:** Stefanie E. M. van Opstal, Marlies N. Wagener, Harald S. Miedema, Elisabeth M. W. J. Utens, Femke K. Aarsen, Linda C. van der Knaap, Eric C. M. van Gorp, Annemarie M. C. van Rossum, Pepijn D. D. M. Roelofs

**Affiliations:** 1 Erasmus MC, Department of Viroscience, University Medical Center Rotterdam, Rotterdam, The Netherlands; 2 Centre of Expertise Innovations in Care, Rotterdam University of Applied Sciences, Rotterdam, The Netherlands; 3 Erasmus MC, Department of Child and Adolescent Psychiatry, University Medical Center Rotterdam, Rotterdam, The Netherlands; 4 The Bascule, Academic Medical Centre, University of Amsterdam, Amsterdam, The Netherlands; 5 Princess Maxima Center for Pediatric Oncology, Utrecht, The Netherlands; 6 Erasmus MC, Sophia Children’s Hospital, Rotterdam, The Netherlands; University of Perugia, ITALY

## Abstract

**Introduction:**

Since the introduction of combination antiretroviral therapy, human immunodeficiency virus (HIV) infection is a manageable chronic disease. However, school-age children (4–18 years) living with HIV could still experience problems with functioning at school, due to the impact of the virus itself, medication, comorbidities and social stigma. School functioning covers academic achievement, school attendance, and social relationships and is of utmost importance to optimize normal participation.

**Methods:**

To gain insight in school functioning problems of perinatally HIV-infected children, we performed a systematic review of the literature in multiple databases from January 1997 up to February 2019. Studies were included if they described outcomes of school functioning of school-age children perinatally infected with HIV, in high-income countries. Meta-analyses were performed for sufficiently comparable studies.

**Results and discussion:**

Results from 32 studies show that HIV-infected children experience more problems in various areas of school functioning in comparison with national norms, matched healthy controls, siblings and HIV-exposed uninfected (HEU) children. The most pronounced differences concerned the usage of special educational services, general learning problems, and mathematics and reading performance scores. Comparisons with both national norms and siblings/HEU children show that the differences between HIV-infected children and siblings/HEU children were less pronounced. Moreover, siblings/HEU children also reported significantly worse outcomes compared to national norms. This suggests that problems in school functioning cannot be solely attributed to the HIV-infection, but that multiple socio-economic and cultural factors may play a role herein.

**Conclusion:**

Perinatally HIV-infected children seem vulnerable to problems in various areas of school functioning. Therefore, monitoring of school functioning should be an important aspect in the care for these children. A family-focused approach with special attention to a child’s socio-environmental context and additional attention for siblings and HEU children, is therefore recommended.

## Introduction

The introduction of combination antiretroviral therapy (cART) in 1996 has dramatically decreased the mortality and morbidity rates of children living with human immunodeficiency virus (HIV) worldwide [1, 2]. Consequently, the daily functioning of children living with HIV has gained greater research interest [3–6].

Nowadays, nearly 2 million children (<15 years) worldwide are living with HIV, and more and more of them reach school age [7]. Despite that viral suppression can be achieved with adequate medication, the daily functioning of children living with HIV is still affected by the social stigma attached to HIV, the high probability of comorbidities, and long-term effects of medication [8, 9]. For children living with HIV, school functioning is of utmost importance, as it provides a sense of normalcy and belonging–and is crucial for developing self esteem and social skills [8, 10, 11]. School functioning is a broad concept that covers academic achievement, school attendance and social relationships at school [10]. Aspects of school functioning can be assessed in many ways, looking at different educational outcomes, such as grade retention or receiving special services at school [5, 11].

Previous studies have described associations between having a chronic disease and many different and interrelated educational outcomes [12, 13]. Having a chronic disease has been associated with more peer problems, lower motivation to do well at school, lower academic achievement, higher levels of special education, and poorer school attendance [12, 14–16]. School attendance is highly important for developing peer relationships and social competence. Conversely, school absenteeism has been associated with lower educational attainment, a higher risk of grade retention, and drop-out [11, 17]. The pathway from a chronic disease towards problems in school functioning is complex, as many factors could play a role herein, such as side effects of medication, illness-induced stress, and lower expectations from family and school [12].

Still, two important factors differentiate HIV from most other chronic diseases with respect to school functioning. One is the social stigma associated with HIV [18]. The second concerns the multigenerational aspect of the infection, in that the child’s parents may be at higher risk for illness and premature death. The impact of parental HIV-related illness and death on children’s school functioning has been described in three previous reviews, which showed disadvantages in various measures of school functioning [5, 19, 20].

Moreover, perinatally HIV-infected children are also at risk for problems in their cognitive, behavioral and emotional functioning [21–23]. Such problems–for example, deficiencies in executive functioning or processing speed, most likely interfere with school functioning outcomes.

In the Netherlands, healthcare providers have raised concerns about the school functioning of children with a perinatally acquired HIV-infection. They have reported problems with these children’s academic performance, social problems with peers at school, and pointed out the need for special education. In high-income countries (HICs) such as the Netherlands, a large majority of children receive free public primary and secondary education, which is compulsory for a specific age-range, e.g. 5–16 years [24–26]. High enrolment ratios and low percentages of out-of-school children are relatively common in HICs [27]. Worldwide, school enrolment ratios and the average duration of schooling have increased considerably [27, 28]. Unfortunately, disparities in the access to education for children still exist, with for instance a net school enrolment rate of 79% in sub-Saharan Africa in 2018, compared with more than 90% worldwide [27]. Furthermore, multidisciplinary care and treatment including cART are usually available in HICs [7, 29]. For example, in the Netherlands, 98% of HIV-infected children receive cART [30]. Worldwide there is a huge disparity in access to healthcare services and antiretroviral therapy; for example, 58% of all people living with HIV in Western and Central Africa have access to cART compared with 81% in Western and Central Europe and North America [31]. Since limited access to education and healthcare services could negatively influence the school functioning of children with HIV, it is important to take these contextual differences into account.

Few previous reviews have presented data on the school functioning of HIV-infected children. Two of these reviews had a broader scope than school functioning alone and presented only limited data on educational outcomes of HIV-infected children. Their focus was on quality of life and developmental challenges of HIV-infected children, resulting in a search strategies with no specific focus on school functioning [4, 32]. Three other previous reviews focused mainly on children affected by parental HIV-related illness and death in low- and middle-income countries (LMICs) [5, 19, 20]. Despite the overlap in vulnerabilities and social and cultural context between HIV-affected and HIV-infected children, the pathways leading to problems in school functioning in these groups are clearly different [20].

To our knowledge, a systematic literature review that specifically focuses on the school functioning of HIV-infected children and comprehensively describes and quantifies their educational outcomes is lacking. Furthermore, no previous reviews have compared school functioning of HIV-infected children with that of HIV-uninfected children and national normative data in meta-analyses.

Therefore, we performed a review of the literature that focuses on school functioning of HIV-infected children in HICs, addressing the following research question: What problems in school functioning are described in current literature for perinatally HIV-infected children in high-income countries? The rationale for this question is that insight in these challenges of perinatally HIV-infected children is needed to be able to facilitate their educational needs.

## Method

### Study design

We performed a systematic review, consisting of a systematic literature search, appraisal and synthesis of available research evidence published from January 1997 up to and including February 2019 [33]. For this systematic review we have followed the PRISMA (Preferred Reported Items for Systematic Review and Meta-analysis guidelines (see S1 Checklist). The literature search was performed in February 2019, the appraisal and synthesis was completed in September 2019. Steps of a review protocol were used as described below.

### Inclusion criteria for this review

#### Types of studies

We included randomized controlled trials (RCTs), controlled clinical trials (CCTs), cohort studies, case-control studies, cross-sectional studies and qualitative studies. Meta-analyses and reviews were excluded, but checked on references to other articles than retrieved in our search.

#### Context of studies

Only studies from high-income countries were included in order to reduce the variability in access to education and healthcare systems. A high-income country is defined as an economy with a gross national income per capita US$12,056 or more in 2017 [34]. Studies were eligible if they reported data collected after 1996, when cART became widely available.

#### Participants

We included studies focusing on perinatally HIV-infected children of school age (4–18 years). Studies in which the primary diagnosis of the participants was not HIV were excluded.

#### School functioning

Studies were eligible if they described school functioning of children with perinatally obtained HIV-infection. Studies that applied the following outcomes of school functioning were included:

- The need for special educational services at school
- Special education
- School absence
- Drop-out of school
- Learning in school
- Academic achievement
- Social participation at school

### Search methods for identification of studies

We searched the following electronic databases for articles published in English: PubMed publisher, PsycINFO OvidSP, CINAHL, Cochrane, Web of Science, Embase, Medline OvidSP, ERIC (ProQuest) and Google Scholar. A sensitive search strategy was performed, included variations and Boolean connection (AND, OR, NEAR) of the key terms (and their synonyms): HIV and school functioning (S1 Appendix). Search terms were chosen by consensus among a group of medical professionals in HIV-treatment and paediatric neuropsychologists, based on existing literature and clinical experience. Reference lists of relevant articles were hand searched to identify additional relevant publications.

### Data collection and analysis

#### Selection of studies

We used the following process for selection of studies:

- We merged search results and removed duplicates using reference management software Endnote ®.
- Two review authors (SO and MW) independently screened the titles and abstracts of the retrieved citation against the criteria mentioned above and removed clearly irrelevant records.
- Full-text articles were collected and assessed for eligibility on the basis of the inclusion criteria by SO and MW.
- SO and MW independently extracted from each included article data on study aim, study design, study population and sample size, control group, method and key findings related to our research question.
- A third reviewer (PR) was consulted if any disagreements regarding selection of relevant studies or data extraction occurred.

#### Assessment of risk of bias in included studies

Two authors (SO and MW) separately performed a quality assessment of included studies at study level, using the various assessment forms of the Dutch Cochrane Center and the Scottish Intercollegiate Guidelines Network (SIGN) [35, 36]. A third reviewer (PR) was consulted in case of disagreement regarding the methodological quality assessment.

#### Data synthesis and analysis

We performed a meta-analysis if studies were sufficiently comparable, judged on similarity in setting, control group, participants and type of outcome. Review Manager version 5.3 was used for the analyses [37]. Only studies that compared outcomes of school functioning with a control group were included in the meta-analyses. Dichotomous outcomes were analyzed by calculating the relative risk (RR). We interpreted the effect size of the RR as high, when the RR was ≤0.50 or ≥1.50, and as moderate when >0.50 but ≤0.75 or <1.50 but ≥1.25 [38]. We analyzed continuous outcomes by calculating the mean difference (MD) when the same instrument was used to measure outcomes, or the standardized mean difference (SMD) when different instruments were used (Cochrane Handbook version 5.1.0). Uncertainty was expressed with 95% confidence intervals. We used a random-effects model to incorporate heterogeneity among studies [39]. We performed subgroup analyses with either the national reference population or siblings and HIV-exposed uninfected (HEU) children as control group. Interpretation of the effect size of continuous outcome measures with SMD was based on Cohen´s method: small when SMD >0.20 but <0.50; moderate when SMD ≥0.50 but <0.80; and large when SMD ≥0.80 [40]. When we refer to significant differences in the results, we mean statistically significant with a p-value ≤0.05. A narrative synthesis was performed summarizing the different aspects of school functioning if meta-analysis was not possible.

## Results

**Fig 1:**
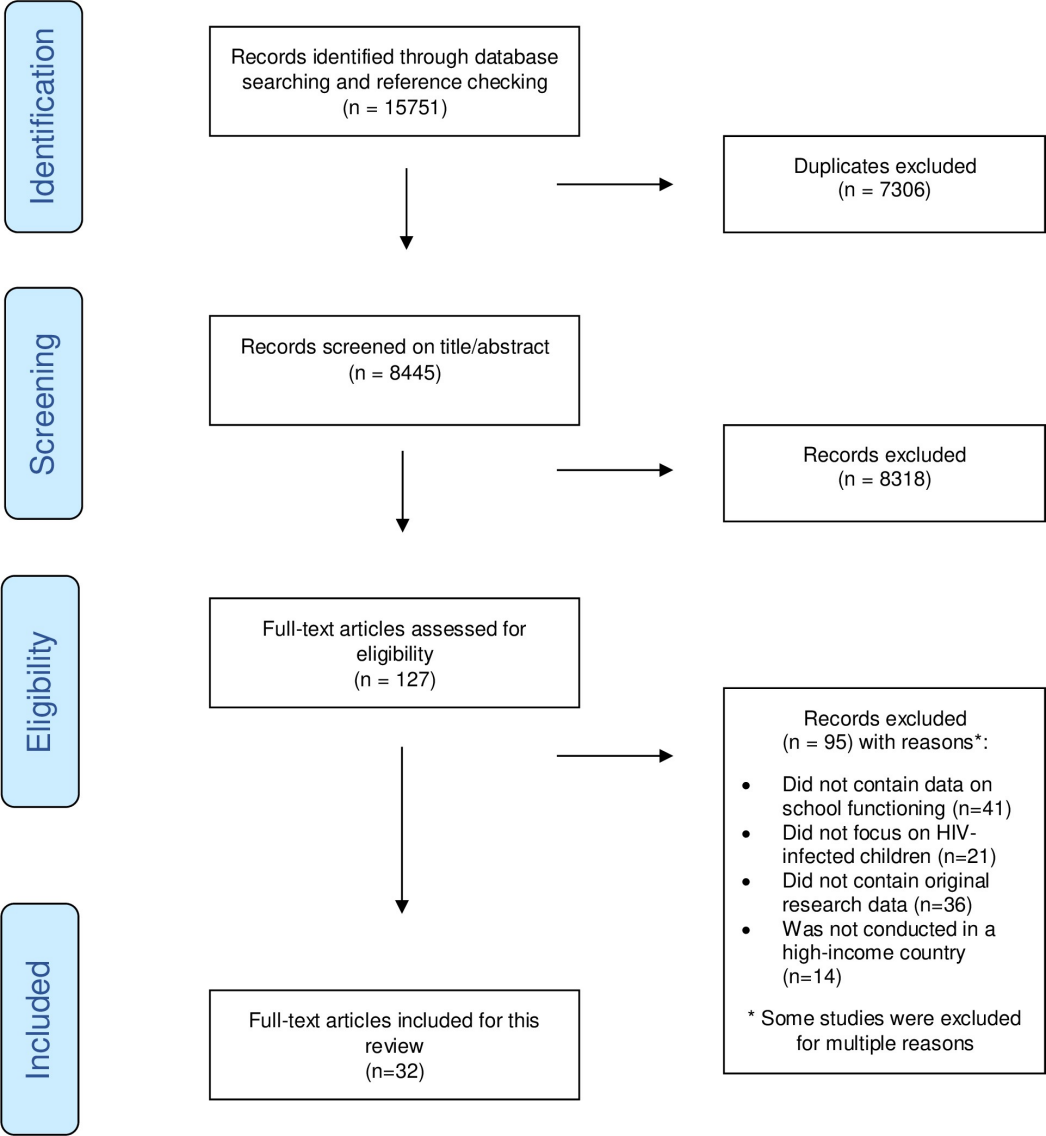
Selection of eligible studies.

The search strategy identified 8445 original publications after removal of duplicates. Most of the publications that did not meet the inclusion criteria focused on education about HIV at school. Eventually, 32 publications met the inclusion criteria. The selection process is presented in Fig 1. Below, we first describe the quality assessment and the general study characteristics of the 32 included studies; next the outcomes of school functioning.

### General study characteristics

The results of the quality assessment of the studies are presented in S2 Appendix. Overall, the quality of the included studies was high, as the majority of criteria regarding methodological quality were met, with little risk of bias. The most frequent flaw was neither reporting the confidence intervals (CI) nor the standard deviations (SD). We used descriptive data from the studies showing this flaw, and the results of those studies were not included in meta-analyses.

Most studies had been performed in the USA (n = 25), followed by Italy (n = 2), Spain (n = 2), Canada (n = 1), France (n = 1) and the Netherlands (n = 1). Study designs were: cohort (n = 10) [3, 41–49]; cross-sectional (n = 20) [6, 50–68] and qualitative (n = 2) [8, 69]. Of the ten cohort studies, four measured the school functioning outcomes only once and two presented only baseline data of school functioning; for this review they were therefore labelled as cross-sectional [3, 41, 42, 45, 46, 49]. Two other cohort studies investigated the change in school functioning after a modification in medication. Of these two studies we used the baseline outcomes of school functioning [44, 47]. Studies with quantitative data compared the outcomes of school functioning of HIV-infected children with national norms (n = 15) [3, 6, 41, 43–45, 47, 51–53, 59, 60, 64, 67, 68], matched healthy controls (n = 3) [50, 53, 54], HIV-exposed uninfected children (n = 5) [46, 52, 57, 59, 68] or siblings or other children living in the same household (n = 4) [6, 55, 56, 58].

Definitions and methods to investigate school functioning were heterogeneous among the studies. Table 1 provides an overview of types of outcome and applied instruments. Studies described or evaluated: the need for special educational services at school, special education, school attendance, drop-outs, learning and social participation at school. In addition, several studies evaluated reading, spelling and mathematic achievement as a way to assess academic achievement [6, 43, 51, 52, 54, 59, 68]. Furthermore, eight studies described the need to repeat a class as a way to evaluate school functioning [3, 45, 48, 51, 52, 61, 62, 65, 66]. Six studies applied a previously validated instrument measuring multiple aspects of school functioning, which were summarized in one general school functioning scale [50, 53, 58, 59, 63, 65]. Other studies evaluated aspects of school functioning through self-designed questionnaires (n = 7) [3, 45, 54–56, 61, 62], reviews of medical records (n = 6) [42, 48, 49, 62, 67, 69] or interviews (n = 5) [8, 51, 52, 66, 69]. See S3 Appendix for an overview of general study characteristics and key findings per study.

**Table 1:** Overview of school functioning outcomes and applied instruments.

*What is measured*?	*Instrument*	*Content of instrument/measurement*	*Articles*
**Need for special education and special educational services**	-	Percentages of children with and without HIV in full time special education or using special educational services.	Brackis-Cott et al., 2009b Cohen et al., 2015 Dolfus et al., 2010 Gadow et al., 2010 Malee et al., 2011 Medin et al., 2016 Mialky et al., 2001 Storm et al., 2005 Wolf et al., 2016
**Repeating classes**	-	Percentages of children with and without HIV who had to repeat a class.	Brackis-Cott et al., 2009a Brackis-Cott et al., 2009b Dolfus et al., 2010 González-Tomé et al., 2018 Malee et al., 2011 Medin et al., 2016 Mialky et al., 2001 Storm et al., 2005 Wolf et al., 2016
**School attendance and drop-outs**	-	Percentages of children with and without HIV who dropped out of school or completed high school.	Battles et al., 2002 Brackis-Cott et al., 2009b Vuppula, et al., 2017
**Reading, Spelling and Mathematics**	Wide Range Achievement Test	Achievement test measuring: • Reading recognition • Spelling • Arithmetic computation (mathematics)	Blanchette et al., 2002 Brackis-Cott et al., 2009a Brackis-Cott et al., 2009b
	Woodcock Johnson Test of Achievement	Achievement test measuring: • Passage comprehension (reading) • Spelling • Applied problems (mathematics)	Franklin et al., 2007
	Wechsler Individual Achievement Test	Achievement test measuring: • Word reading • Spelling • Numerical operations (mathematics)	Garvie et al., 2014 Sirois et al., 2016b
	End-of-Grade test	Achievement test measuring: • Reading • Mathematics	Ellis, 2004
**Learning**	Conners’ Parent Rating Scales, Learning subscale	Questionnaire for caregivers regarding: • Spelling • Understanding and speed of reading • Memorizing facts and lessons learned • Understanding of instructions • Learning information as separate facts • Understanding arithmetic	Jeremy et al., 2005 Kullgren et al., 2004 Malee et al., 2011 Mellins et al., 2003 Nozyce et al., 2006 Sirois et al., 2016a
**General school functioning**	Child Behavior Checklist, School competence scale	Questionnaire for caregivers regarding: • Performance on academic subjects • Special educational services, special class or special school • Repeating grades • School problems in the last 6 months	Bomba et al., 2010
	Pediatric Quality of Life Inventory, School functioning scale	Questionnaires for caregivers and children/adolescents regarding: • attention ability in class • memory • difficulty to follow classroom activities • missing school because of not feeling well • missing school to go to the doctor or hospital	Bomba et al., 2010 Cohen et al., 2015
	Wechsler Individual Achievement Test, Total Academic Achievement	Achievement test measuring: • Total Academic Achievement including the word reading, spelling and numerical operation subscale.	Garvie et al., 2014
	Social and Academic Functioning Questionnaire, School Functioning subscale	Questionnaire for caregivers regarding: • Mean performance in academic subjects • Behavior problems; suspensions and other disciplinary actions • Grade retention • Special and remedial teaching	Gadow et al., 2010 Nachman et al., 2012
	National Health Interview Survey, Social and School functioning subdomain	Questionnaire for caregivers regarding: • Repeating a grade • Receiving special school help • Health limited school attendance • Health limited activities • ≥1 bed day in 4 weeks	Storm et al., 2005
**Social participation at school**	Social Support Scale for Children	Questionnaire for children regarding their experiences with: vClassmate support: the extent to which one’s classmates like them, are friendly, don’t make fun of them, listen and ask them to join in play • Teacher support: the extent to which one’s teacher help them if they are upset, help them to do their best, care about them are fair to them and treat them as a person.	Battles et al., 2002

### School functioning

#### Need for special education and special educational services

**Fig 2:**
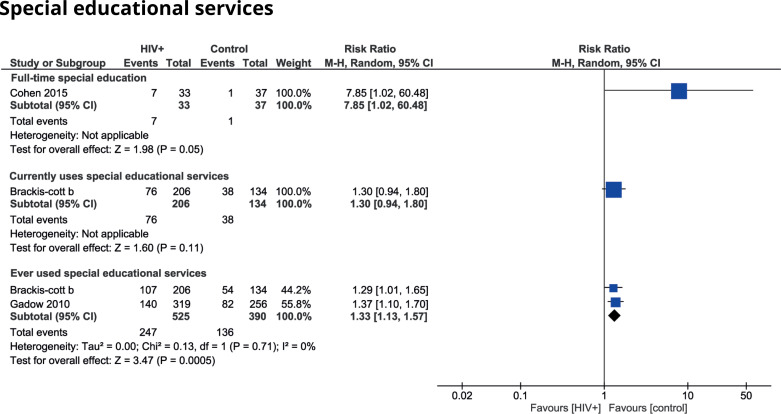
Special educational services used by HIV-infected children compared to control groups.

Ten studies reported the need for special education or special educational services for children with HIV [3, 45, 48, 49, 52, 53, 58, 61, 62, 65]. The weighted average percentage for the need for special educational services at school is 35.7% [45, 61, 62, 65] and that for full-time special education is 7.4% [3, 48, 53, 62]. Three studies made a comparison between children with HIV and a control group [52, 53, 58]. One study made a comparison with healthy controls matched on socioeconomic status, gender, ethnicity and age and found a significantly higher chance of being in full-time special education for children with HIV [53]. The two other studies made a comparison with siblings and HEU children. Both studies found a significantly higher chance of the usage of special educational services in the past in the HIV-group. One reported in addition a higher, but not significant probability of the current usage of special educational services for HIV-infected children. A meta-analysis was only possible for the usage of special education in the past and shows a moderate effect size (RR 1.33; 95% CI 1.13 to 1.57). Regarding the other outcomes, a meta-analysis was not possible because only one study made use of a comparison group (Fig 2). Overall, a trend exists towards an increased usage of special education and special educational services for HIV-infected children.

#### Repeating classes

Repeating classes by HIV-infected children was addressed in nine studies [3, 45, 48, 51, 52, 61, 62, 65, 66]. The proportion of children with HIV repeating at least one grade ranged from 21% till 61%, with a weighted average of 29%. Two of these studies compared this proportion with that of respectively a control group of HEU children and national normative data [3, 51]. Both found that the proportion of HIV-infected children repeating classed was higher, but not statistically significantly higher, than the respective proportion of controls or the national norm. A meta-analysis was not possible because the nature of the control groups differed.

#### School attendance and drop-outs

Six studies described school attendance and drop-out by HIV-infected children [3, 41, 45, 54, 61, 65]. Three studies made a comparison with a control group [41, 52, 67]. Results were mixed: Brackis-Cott et al. (2009b) found no difference between HIV-infected children and HEU children. Battles et al. (2002) reported significantly more drop-outs compared to national norms. In contrast, Vuppula et al. (2017) reported that significantly more children living with HIV completed high school compared to students from the same city. A meta-analysis was not possible because of different control groups in the three studies.

#### Reading, spelling, and mathematics

**Fig 3:**
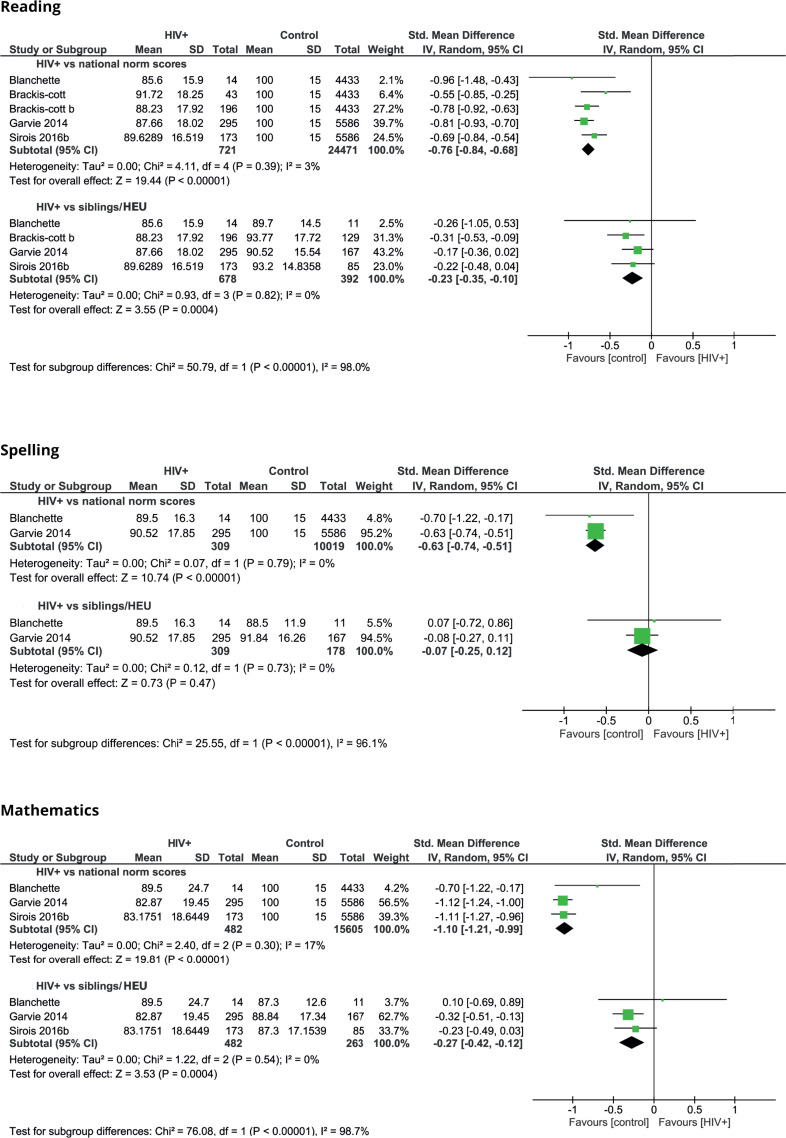
Reading, spelling and mathematic scores of children with HIV compared to control groups.

Academic achievement was in many studies evaluated by measuring the ability in reading, spelling and mathematics (Fig 3). Five studies made a comparison of children with HIV and the national reference population regarding reading and found significant differences in favour of the national reference population, with an overall moderate effect size (SMD -0.76; 95% CI -0.84 to -0.68) [6, 51, 52, 59, 68]. Two studies also considered spelling and found significant differences in favour of the national reference population, with a moderate effect size (SMD -0.63; 95% CI -0.74 to -0.51). Four studies considered mathematics and found significant differences in favour of the national reference population, with a large effect size (SMD -1.10; 95% CI -1.21 to -0.99). Two other studies described the same trend in comparison with national norms regarding reading, spelling and mathematics, but did not report the exact numbers and statistical significance [43, 54].

Four studies made a comparison of HIV-infected children and siblings or HEU children [6, 52, 59, 68]. With regard to reading, the overall effect size was small (SMD -0.23; 95% CI -0.35 to -0.10) in favour of siblings and HEU children. Two of these studies also considered difficulties in spelling, but overall no significant difference was found. Three studies also considered mathematics, of which one found a significant difference. Overall, the effect size was small (SMD -0.27; 95% CI -0.42 to -0.12) in favour of siblings and HEU children. Two studies reported that difficulties in reading and mathematics were the main reason for HIV-infected children for attending a special education class [51, 52].

#### Learning problems

**Fig 4:**
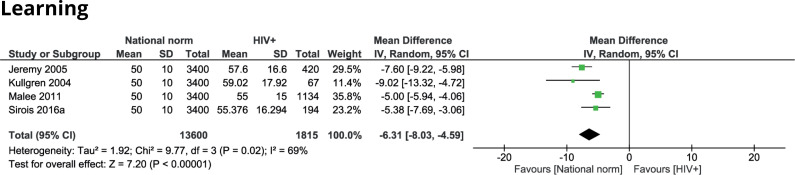
Learning scores of HIV-infected children compared to national norm scores.

Five studies applied the Conners’ Parent Rating Scale (CPRS) to evaluate learning problems in HIV-infected children [44–47, 60]. Four of these studies made a comparison with national norms and found significant differences in favour of the national reference population [44, 45, 47, 60]. Overall, the HIV-infected children were assigned a score -6.31 (95% CI -8.03 to -4.59) lower than the national norms (Fig 4). This difference corresponds with a moderate effect size (SMD -0.61; CI 95% -0.79 to -0.44). One study compared learning problems measured with the CPRS between children with HIV and HEU children and found no significant difference [46].

#### General school functioning

Six cross-sectional studies applied a previously validated instrument measuring multiple aspects of school functioning, which were summarized in one general school functioning scale [50, 53, 58, 59, 63, 65]. Four of these studies made comparisons between children with HIV and control groups [50, 53, 58, 59] (S4 Appendix). Two studies included a control group of respectively HEU children and children living in the same household and found significantly more problems for children with HIV [58, 59]. The two other studies included matched healthy controls, of which one found significantly more problems among children with HIV [50]. Two studies made an additional comparison with national norm scores; both reported significantly more problems for children with HIV [53, 59]. A meta-analysis was not performed because the instruments used to measure general school functioning were too heterogeneous and the nature of the control groups differed. In addition, two studies investigated the development of school functioning over time, of which one found no significant differences and one described a trend towards deterioration [43, 48].

#### Social participation at school

Eight studies reported on social participation of HIV-infected children at school [8, 41, 54–56, 61, 62, 69]. Two studies reported the proportion of children who had disclosed their HIV-status at school, which was 17% and 24% respectively [61, 62]. Two qualitative studies reported about stigma and non-disclosure at school [8, 69]. The children who disclosed their HIV-status most often did this to a school nurse or teacher only. Five studies described social problems in the classroom for HIV-infected children, such as taunting, involvement in fistfights and disruptive behavior [8, 54–56, 69]. None of these studies made a comparison with a control group.

## Discussion

This review is the first to focus specifically on the school functioning of perinatally HIV-infected children in high-income countries (HICs). Moreover, we compared school functioning of HIV-infected children with that of control groups in meta-analyses. We conclude that children with HIV living in a HIC experience more problems in various areas of school functioning compared to national norms, matched healthy controls, siblings and HEU children. The most pronounced differences concern the usage of special educational services, reading and mathematics performance scores, and general learning problems.

Several included studies, found that HIV-infected children have significantly more problems in various areas of school functioning when compared with siblings and HEU children. It must be noted, however, that siblings and HEU children themselves scored significantly worse on multiple school functioning outcomes when compared to national norms. This suggests that the problems in school functioning cannot be solely attributed to the HIV-infection, but that multiple social factors may play a role herein, such as parental drug abuse, lack of social support, parental illness and death, or changes in living circumstances. These factors are common among HIV-infected children as well as their siblings or HEU children and appear to have a substantial impact on school functioning of all these groups [18, 41, 69]. Previous studies confirm the negative influence of parental HIV-infection on children’s school functioning [5, 70]. Pathways from parental HIV-infection to school problems include psychological distress of the child, poorer school attendance instigated by the need to help at home, limited supervision of homework, and HIV-related discrimination by classmates [70, 71]. Low socioeconomic status (SES) and living in an impoverished neighbourhood could further complicate school functioning of HIV-infected children and those affected by HIV due to parental HIV [72]. In HICs, the relation between SES and school functioning is well established. Family SES influences, for example, the kind of school and classroom environment to which a child has access to, as well as the physical resources, such as a fixed place in the home for studying, and social resources, such as the amount of supervision over homework [73]. In resource-rich settings, HIV generally emerges in vulnerable, sometimes marginalized sub-populations, such as people who inject drugs or migrants from low- and middle-income countries (LMICs). In HICs, the majority of HIV-infected children are represented by racial and ethnic minorities. For example, in the United States, the Centers for Disease Control and Prevention (CDC) reported that Black/African American children are disproportionately affected by HIV, and accounted for 65% of the new diagnoses of perinatal HIV-infections in 2018 [74]. In the Netherlands, the proportion of HIV-infected children with two native Dutch parents is less than 3%, and 57% of the under 18-year-old HIV-infected children were born in sub-Saharan Africa [30]. Having migrated from an LMIC to a HIC has been associated with an disproportionate risk of acquiring HIV-infection, through a combination of factors, including stigma and disruption of one’s social network [75]. Regarding racial and ethnic disparities in school functioning, a review revealed that the academic performance of students from underrepresented minorities could lag behind that of other students [76]. Another review found a relation between low SES and academic problems in immigrant children and youth in Europe [77]. When reviewing school functioning of HIV-infected children in HICs, it is therefore important to acknowledge the influence of sociodemographic and contextual differences, for instance by relating their school function to that of siblings.

Apart from the possible effects of the aforementioned factors, it is unclear whether there is a direct relation between HIV-infection status and school functioning. Some studies have indicated an association between poor school functioning and AIDS-defining conditions, such as previous poor viral suppression or opportunistic infections [42, 59, 65]. Moreover, HIV-infection status has been associated with neurocognitive problems. Before cART was available, the prevalence of HIV-encephalopathy in perinatally HIV-infected children with AIDS was estimated at 30–50% [78, 79]. Prior HIV-encephalopathy has been significantly associated with poor school functioning [42, 59]. After the introduction of cART in 1996, a 10-fold decline in the incidence of HIV-encephalopathy was observed [80]. Nonetheless, neurocognitive deficits remain present in HIV-infected children on long-term cART, which could have a negative impact on school functioning [80, 81].

Regardless of the exact mechanism that relates having a perinatally acquired HIV-infection to school functioning, evidence suggests that HIV-infected children are at risk of experiencing problems at school and failing to meet their educational potential. Poor school functioning can diminish job opportunities and thus lead to a greater probability of unemployment in the future [82]. Problems with school functioning could furthermore compromise the health status of HIV-infected children. Low educational attainment has been associated with problems in medical treatment adherence among adolescents with HIV [83]. Reading problems in particular could have implications for the ability to understand one’s illness and for the adherence to challenging medical regimens [52]. Furthermore, the difficulties in school functioning were often accompanied by emotional and behavioral problems. This circumstance has been supported by numerous studies included in this review, which describe both increased emotional and behavioral problems and problems in school functioning in children with HIV [43–46, 50, 62]. To note, in these studies, younger children generally were not aware of their HIV-status. Two studies reported that respectively 38% and 43% of the total study population knew their HIV-status [45, 62]. It is well possible, therefore, that concurrent the emotional and behavioral problems are only partly associated with the stress of living with a stigmatized illness.

In the included studies, many different measurement instruments were used to assess aspects of school functioning, such as the End-of-Grade test to measure reading and mathematics (Table 1). It is important to note that such instruments might not capture the whole picture of school functioning. Only four of the included studies conducted interviews with HIV-infected children regarding their school functioning to gain a comprehensive understanding of the actual experienced challenges of HIV-infected children [8, 51, 52, 66]. Studies measuring multiple educational outcomes and in addition focusing on the experiences of HIV-infected children in school are therefore recommended.

Most of the included studies covered a broad age range and focused on both younger children and adolescents. A minority of the included studies (5/32) explored the possible association of age with educational outcomes [43, 45, 52, 59, 64]. In four out of these five studies, a significant association was found between older age and more problems in school functioning [45, 52, 59, 64]. In addition, five studies explored gender differences in relation to educational outcomes [3, 46, 52, 53, 64]. Only one of these found a significant association between gender and school functioning [46]. Considering that HIV can affect school functioning differently across age groups and gender, we recommend further research to explore these associations.

Strengths of this review are the sensitive search and the broad inclusion criteria regarding educational outcomes. Nonetheless, this also yields some limitations. Many of the included studies did not primarily focus on school functioning, but rather on quality of life or neuropsychological functioning of HIV-infected children. School functioning was therefore often a secondary outcome; for example, some studies provided only basic statistics of children in special education or dropping out of school, without mentioning reasons. The fact that most of the included studies were performed in the USA (78%) and Europe (19%), restricts generalizability to all HICs. Regarding the quality assessment of the included studies, some studies had small sample sizes, did not provide confidence intervals or lacked a control group, which made it difficult to define if there were actual problems in school functioning. In addition, the definition of school functioning varied among the included studies, and this resulted in heterogeneity of applied instruments. Moreover, the study populations varied with respect to medical regimen, current viral suppression and cultural background. However, despite this heterogeneity in definitions, measurements and populations, the results are overall consistent, suggesting a general disadvantage in the area of school functioning for HIV-infected children. Moreover, the overall methodological quality of the included studies was good, which makes the risk of bias small and our results plausible.

By selecting studies from HICs, we aimed at reducing the variability in access to education and healthcare systems. We assumed that in HICs most children have access to cART and consequently have a good viral suppression. This assumption proved accurate; most of the children in the included studies indeed received cART. However, information regarding the exact medical regimens was lacking in some of the included studies, although it was clear that the studied children received medical care in specialized healthcare facilities. Furthermore, some studies included also respondents who did not use cART, for instance due to problems with adherence. Many studies also included respondents born before cART became available, which might have negatively influenced the cognitive development in their early lives.

With regard to healthcare, impeccable achievements have been made in LMICs in increasing access to adequate healthcare services and providing HIV-infected children with cART. Nevertheless, in 2019, only 53% of HIV-infected children (aged 0–14 years) worldwide were receiving adequate treatment with cART [7]. Access to healthcare services, and cART in particular, influences the HIV-related morbidity and mortality rates. Consequently, HIV-infected children living in a context of limited access to healthcare services, including cART, are at higher risk to suffer from the impact of HIV-related illness on their school functioning. Furthermore, parental illness and death due to a lack of medication, could place perinatally HIV-infected children at additional risk to be forced into child labour or take responsibility for their siblings in a child-headed household [84]. In LMICs, illness or death of a parent due to HIV-infection often leads to an economic decline of family income, which could result in problems with paying school fees. Decreased school attendance and high rates of drop-outs are therefore not uncommon for HIV-infected children in LMICs [85].

Regarding education, the number of out-of-school children is globally decreasing, although some regions are still facing challenges in the access to education. For instance, in West and Central Africa, nearly one quarter of the younger children were out of primary school in 2018 [27]. Worldwide, there is also still a disparity in the average duration of education received by people aged 25 years and over, which was 5.8 years in Sub-Saharan Africa, compared to 10.4 years in Europe in 2019 [27].

Thus, despite worldwide improvements, there are still disparities in access to education and healthcare services, including cART, which could negatively impact the school functioning of HIV-infected children. Therefore, this review has focused on studies conducted in HICs, where access to good healthcare facilities, including multidisciplinary care and provision of cART, are prevalent and where the percentages of out-of-school children are very low [7, 27, 28]. As a consequence, the results are primarily valid for the situation in HICs. However, the insights in school functioning of HIV-infected children of this review may also be important for LMICs, especially when taking into account the impact of access to education and healthcare services.

## Conclusion

In this review we found that perinatally HIV-infected children are vulnerable to problems in various areas of school functioning. Therefore, the monitoring of school functioning should be an important aspect in the care for these children. The relatively small differences in school functioning between HIV-infected children and their siblings or HEU children suggest that part of the problems in school functioning of HIV-infected children can be explained by their socio-economic and cultural circumstances. A family-focused approach, addressing special attention to the socio-environmental context of the children, is therefore recommended. As siblings and HEU children face partly the same problems in school functioning, they should also receive extra attention. HIV-infected children mostly have regular contact with medical, mental health and social service professionals, which could help them receive the services they need. Their siblings and HEU children are less likely to benefit from these services. As HIV-infection is still a stigmatized disease, the HIV-status is often not disclosed at school. Therefore, it is important that medical professionals are aware of the compromised school functioning of children with HIV and help the parents, children with HIV and their siblings navigate systems to receive the services they need.

## Supporting information

S1 Checklist PRISMA 2009 checklist. (DOC)

S1 Appendix Search strategy. (DOCX)

S2 Appendix Quality assessment of included studies. (DOCX)

S3 Appendix General study characteristics and key findings per study. (DOCX)

S4 Appendix General school functioning scores. (DOCX)
