# Supplementary material for: School functioning of children with perinatal HIV-infection in high-income countries: A systematic review
Source: PLoS One. 2021 Jun 4;16(6):e0252746. doi: 10.1371/journal.pone.0252746 (PMC8177442; doi:10.1371/journal.pone.0252746)
Supplement: S1 Appendix — (DOCX) [file pone.0252746.s002.docx]

# S1 Appendix. Search Strategy

**Embase**

('Human immunodeficiency virus infection'/exp OR 'Human immunodeficiency virus'/exp OR 'Human immunodeficiency virus infected patient'/de OR (hiv OR ((immunodeficiency OR 'immuno deficiency') NEXT/1 (virus* OR syndrom*)) OR seropositiv* ):ab,ti) AND ('academic achievement'/exp OR 'school reentry'/de OR ((school/exp OR student/exp) AND (absenteeism/de OR 'peer group'/de)) OR ((school OR education* OR academ* OR learn* OR student* OR classroom OR 'class room' OR universit*) NEAR/3 (participat* OR perform* OR achieve* OR functioning* OR attain* OR absen* OR difficult* OR problem* OR fail* OR habit* OR dropout* OR attend* OR issue* OR grade* OR outcome* OR inadequate OR stigma OR disab* OR cognit* OR behav* OR adapt* OR accept* OR friend* OR fellow* OR pupil* OR need* OR placement* OR success*)):ab,ti) NOT (Africa/exp )

**Medline OvidSP**

(exp "HIV Infections"/ OR exp "hiv"/ OR (hiv OR ((immunodeficiency OR "immuno deficiency") ADJ (virus* OR syndrom*)) OR seropositiv* ).ab,ti.) AND (((exp schools/ OR exp Students/) AND (absenteeism/ )) OR "Student Dropouts"/ OR ((school OR education* OR academ* OR learn* OR student* OR classroom OR 'class room' OR universit*) ADJ3 (participat* OR perform* OR achieve* OR functioning* OR attain* OR absen* OR difficult* OR problem* OR fail* OR habit* OR dropout* OR attend* OR issue* OR grade* OR outcome* OR inadequate OR stigma OR disab* OR cognit* OR behav* OR adapt* OR accept* OR friend* OR fellow* OR pupil* OR need* OR placement* OR success*)).ab,ti.) NOT (exp Africa/ )

**Cochrane**

((hiv OR ((immunodeficiency OR 'immuno deficiency') NEXT/1 (virus* OR syndrom*)) OR seropositiv*):ab,ti) AND (((school OR education* OR academ* OR learn* OR student* OR classroom OR 'class room' OR universit*) NEAR/3 (participat* OR perform* OR achieve* OR functioning* OR attain* OR absen* OR difficult* OR problem* OR fail* OR habit* OR dropout* OR attend* OR issue* OR grade* OR outcome* OR inadequate OR stigma OR disab* OR cognit* OR behav* OR adapt* OR accept* OR friend* OR fellow* OR pupil* OR need* OR placement* OR success*)):ab,ti) NOT (Africa):ab,ti

**Web-of-science**

TS=(((hiv OR ((immunodeficiency OR "immuno deficiency") NEAR/1 (virus* OR syndrom*)) OR seropositiv*)) AND (((school OR education* OR academ* OR learn* OR student* OR classroom OR "class room" OR universit*) NEAR/3 (participat* OR perform* OR achieve* OR functioning* OR attain* OR absen* OR difficult* OR problem* OR fail* OR habit* OR dropout* OR attend* OR issue* OR grade* OR outcome* OR inadequate OR stigma OR disab* OR cognit* OR behav* OR adapt* OR accept* OR friend* OR fellow* OR pupil* OR need* OR placement* OR success*))) NOT (Africa ))

**PsycINFO OvidSP**

(exp "HIV"/ OR exp "aids"/ OR (hiv OR ((immunodeficiency OR "immuno deficiency") ADJ (virus* OR syndrom*)) OR seropositiv* ).ab,ti.) AND ((exp schools/ AND absenteeism/ ) OR exp "School Dropouts"/ OR "School Attendance"/ OR ((school OR education* OR academ* OR learn* OR student* OR classroom OR "class room" OR universit*) ADJ3 (participat* OR perform* OR achieve* OR functioning* OR attain* OR absen* OR difficult* OR problem* OR fail* OR habit* OR dropout* OR attend* OR issue* OR grade* OR outcome* OR inadequate OR stigma OR disab* OR cognit* OR behav* OR adapt* OR accept* OR friend* OR fellow* OR pupil* OR need* OR placement* OR success*)).ab,ti.) NOT (Africa.ab,ti. )

**CINAHL**

(MH "HIV Infections+" OR MH "HIV-Infected Patients+" OR MH "Human Immunodeficiency Virus+" OR (hiv OR ((immunodeficiency OR "immuno deficiency") N1 (virus* OR syndrom*)) OR seropositiv* )) AND (((MH schools+ OR MH students+) AND (MH absenteeism+ )) OR ((school OR education* OR academ* OR learn* OR student* OR classroom OR "class room" OR universit*) N3 (participat* OR perform* OR achieve* OR functioning* OR attain* OR absen* OR difficult* OR problem* OR fail* OR habit* OR dropout* OR attend* OR issue* OR grade* OR outcome* OR inadequate OR stigma OR disab* OR cognit* OR behav* OR adapt* OR accept* OR friend* OR fellow* OR pupil* OR need* OR placement* OR success*))) NOT (MH Africa+ )

**ERIC (Ovid)**

(exp "Acquired Immunodeficiency Syndrome (AIDS)"/ OR (hiv OR ((immunodeficiency OR "immuno deficiency") ADJ (virus* OR syndrom*)) OR seropositiv* ).ab,ti.) AND (((exp Educational Status Comparison/ OR exp Academic Achievement/) AND (absenteeism/ )) OR "Student Dropouts"/ OR ((school OR education* OR academ* OR learn* OR student* OR classroom OR 'class room' OR universit*) ADJ3 (participat* OR perform* OR achieve* OR functioning* OR attain* OR absen* OR difficult* OR problem* OR fail* OR habit* OR dropout* OR attend* OR issue* OR grade* OR outcome* OR inadequate OR stigma OR disab* OR cognit* OR behav* OR adapt* OR accept* OR friend* OR fellow* OR pupil* OR need* OR placement* OR success*)).ab,ti.) NOT (exp Developing Nations/ OR ((africa).ab,ti))

**PubMed publisher**

(hiv[tiab] OR immunodeficiency virus*[tiab] OR immunodeficiency syndrom*[tiab] OR immuno deficiency virus*[tiab] OR immuno deficiency syndrom*[tiab] OR seropositiv*[tiab] ) AND (school[tiab] OR education*[tiab] OR academ*[tiab] OR learn*[tiab] OR student*[tiab] OR classroom[tiab] OR class room[tiab] OR universit*[tiab]) AND (participat*[tiab] OR perform*[tiab] OR achieve*[tiab] OR functioning*[tiab]) NOT (Africa[tiab]) AND publisher[sb]

**Google Scholar**

Hiv "school|educational|academic|learning|classroom|university participation|performance|achievements|achievement|functioning|attainment|absence|difficulties|problems|failure|dropout|dropouts|attendance|issues|grades|success" -africa
