# Supplementary material for: School functioning of children with perinatal HIV-infection in high-income countries: A systematic review
Source: PLoS One. 2021 Jun 4;16(6):e0252746. doi: 10.1371/journal.pone.0252746 (PMC8177442; doi:10.1371/journal.pone.0252746)
Supplement: S2 Appendix — (DOCX) [file pone.0252746.s003.docx]

# S2 Appendix. Quality Assessment of included studies

## Studies with a quantitative design

| Article | Clearly stated research question | Selection respondents | Exclusion Respondents/non-response | Performance bias | Percentage non-response/ drop-out | Comparison participants/ non-participants | Optional Endpoints in cohort clearly defined | Assessors blinded to exposure status | Recognition of detection bias | Clearly defined outcome measures | Clearly stated outcome subjective outcome measures | Exposure level assessed more than once | Identification of confounders | Confidence interval provided |
| --- | --- | --- | --- | --- | --- | --- | --- | --- | --- | --- | --- | --- | --- | --- |
| Battles (2002) |  | N.A. | N.A. | N.A. | 93% (T1)  69.8% (T3) |  |  | N.A. |  |  |  |  |  |  |
| Blanchette (2002) |  |  | N.A. | N.A. | N.A. | N.A. |  |  |  |  |  | N.A. |  |  |
| Bomba (2010 |  |  | N.A. | N.A. | N.A. | N.A. |  |  |  |  |  | N.A. |  |  |
| Brackis-Cott (2009a) |  | N.A. | N.A. | N.A. | N.A. | N.A. |  | N.A. |  |  |  | N.A. |  |  |
| Brackis-Cott (2009b) |  |  | N.A. | N.A. | N.A. | N.A. |  | . |  |  |  | N.A |  |  |
| Chiriboga (2005) |  |  | N.A. | N.A. | N.A. | N.A. |  | N.A. |  |  |  | N.A. |  |  |
| Cohen (2015) |  |  | N.A. | N.A. | N.A. | N.A . |  | N.A. |  |  |  | N.A. |  |  |
| Dolfus (2010) |  | N.A. | N.A. | N.A. | 20,5% died  9,5% lost to follow-up | N.A. |  | N.A. | N.A. |  |  | N.A. . |  |  |
| Ellis (2004) |  | N.A. | N.A. | N.A. | N.A. | N.A. |  | N.A. |  |  |  | N.A. |  |  |
| Ellis (2010) |  |  | N.A. | N.A. | N.A. | N.A. |  | No |  |  |  | N.A. |  |  |
| Ellis (2011) |  |  | N.A. | N.A. | N.A. | N.A. |  | No |  |  |  | N.A. |  |  |
| Franklin (2007) |  | N.A. | N.A. | N.A. | N.A. | N.A. |  | N.A. |  |  |  | N.A. |  |  |
| Fundaro (1998) |  |  | N.A. | N.A. | N.A. | N.A. |  | . |  |  |  | N.A. |  |  |
| Gadow (2010) |  |  | N.A. | N.A. | N.A. | N.A. |  | N.A. |  |  |  | N.A. |  |  |
| Garvie (2014) |  |  | N.A. | N.A. | N.A. | N.A. |  |  |  |  |  | N.A. |  |  |
| González-Tomé (2018) |  |  | N.A. |  | N.A. | N.A. |  | N.A. |  |  |  | N.A. |  |  |
| Jeremy (2005) |  | N.A. | N.A. | N.A. | 86% still participated in week 48 | N.A. |  | N.A. |  |  |  |  |  |  |
| Kullgren (2004) |  | N.A. | N.A. | N.A. | N.A. | N.A. |  | N.A. |  |  |  | N.A. |  |  |
| Malee et al (2011) |  | N.A. | N.A. | N.A. | 67% completed assessments | N.A. |  | N.A. |  |  |  |  |  |  |
| Medin et al (2016) |  | N.A. | N.A. | N.A. | N.A. | N.A. |  | N.A. |  |  |  | N.A. |  |  |
| Mellins (2013) |  |  | N.A. | N.A. | 45% drop-out |  |  | N.A. |  |  |  |  |  |  |
| Mialky (2001) |  | N.A. | N.A. | N.A. | N.A. | N.A. |  | N.A. |  |  |  | N.A. |  |  |
| Nachman (2012) |  | N.A. | N.A. | N.A. | N.A. | N.A. |  | N.A. |  |  |  | N.A. |  |  |
| Nozye (2006) |  | N.A. | N.A. | N.A. | N.A. |  |  | N.A. |  |  |  | N.A. |  |  |
| Sirois (2016a) |  |  | N.A. |  | N.A. | N.A. |  | N.A. |  |  |  |  |  |  |
| Sirois (2016b) |  |  | N.A. | N.A. | N.A. | N.A. |  |  |  |  |  | N.A. |  |  |
| Storm (2005) |  |  | N.A. |  | N.A. |  |  |  |  |  |  | N.A. |  |  |
| Vuppula (2017) |  | N.A. | N.A. | N.A. | N.A. | N.A. |  | N.A. |  |  |  | N.A. |  |  |
| Wolf (2016) |  | N.A. | N.A. | N.A. | N.A. | N.A. |  | N.A. |  |  |  | N.A. |  |  |
| Wood (2009) |  |  | N.A. | N.A. | N.A. |  |  |  |  |  |  | N.A. |  |  |
|  |  |  |  |  |  |  |  |  |  |  |  |  |  |  |
|  |  |  |  |  |  |  |  |  |  |  |  |  |  |  |

*Quality assessment with checklist Cohort Studies of the Scottish Intercollegiate Guidelines Network [36].*

Green: clear description

Yellow: Some parts clear, some parts unclear/ can’t say

Red: Insufficient description/ not present

N.A.= Not Applicable

## Studies with a qualitative design

|  | Clear research question | Adequate method for data collection | Participant selection | Data collection | Audio recording | Data analyses | Research team | Findings consistent and clear |
| --- | --- | --- | --- | --- | --- | --- | --- | --- |
|  |  |  |  |  |  |  |  |  |
| Lichtenstein, 2010 |  |  |  |  |  |  |  |  |
| Rehm, 2000 |  |  |  |  |  |  |  |  |

*Quality assessment with checklist recommended by the Dutch Cochrane Centre [35].*

Green: Clear description,

Yellow: Some parts clear, some parts unclear

Red: Insufficient description
