# Supplementary material for: School functioning of children with perinatal HIV-infection in high-income countries: A systematic review
Source: PLoS One. 2021 Jun 4;16(6):e0252746. doi: 10.1371/journal.pone.0252746 (PMC8177442; doi:10.1371/journal.pone.0252746)
Supplement: S3 Appendix — (DOCX) [file pone.0252746.s004.docx]

# S3 Appendix. General study characteristics and key findings per study

| **Author/ year/location** | **Study aim** | **Design/ method to measure (aspects of) school functioning** | **Study population and sample size** | **Control group** | **Key findings (related to our research question)** |
| --- | --- | --- | --- | --- | --- |
| Battles, H. & Wiener, L.  2002  USA | To examine the psychosocial factors associated with long-term survival of pediatric HIV infection. | Design:  Cohort study  Methods:   - Questionnaires (3 times): SSSC, SPPC/SPPA (self-report), the Parent Questionnaire - Medical records for information regarding school status | N= 55-80  Age: ≥ 8 years  38.9% perinatally infected, 34.7% hemophilia-related transfusion and 26.4% another type of transfusion.  Minimum of 8 years HIV diagnosis prior to first data collection.  Medical treatment unclear | National norms | School attendance and drop-outs:   - 20% of the study population >18 yrs (n=27) dropped out of school, compared to the national norm of 14.1%. - 55% of the study population > 18 yrs (n=27) completed high school graduation, compared to 76.7% of the national norm. |
| Blanchette, N., et al.  2002  Canada | To compare neuro-psychological functioning in school-age children with HIV and a control group of siblings. | Design:  Cross-sectional study  Methods:   - Wide Range Achievement Test (WRAT-R and WRAT-III) | N= 14  Age: 6-14 years  Perinatally infected  Different types of medical treatment  For half of the children there was evidence of moderate or severe suppression of the immune system.  20% maternal substance use during pregnancy for HIV-infected children. | N=11  Age: 6-15 years  Siblings of children with HIV  Population norms | Reading, spelling and mathematics:   - Reading: HIV+: mean 85.6 (SD:15.9), Control group: mean 89.7 (SD:14.5), population norm: mean100 (SD: 15). HIV+ vs control group: p= 0.55. - Spelling: HIV+: mean 89.5 (SD:16.3), Control group: mean 88.5 (SD:11.9), population norm: mean 100 (SD: 15). HIV+ vs control group: p=0.87. - Mathematics (Arithmetic): HIV+: mean 89.5 (SD:24.7), Control group: mean 87.3 (SD:12.6), population norm mean 100 (SD: 15). HIV+ vs control group: p=0.80. - 36% of the HIV+ group and 50% of the control group experienced difficulties in reading, spelling or math compared to 16% of the general population.   General school functioning:   - Mean test scores in all functional areas, except academic achievement were average relative to published norms. - The mean academic scores for both groups fell within the low-average range. |
| Bomba, M., et al.  2010  Italy | To evaluate health-related quality of life, social competence and behavioral problems in children and adolescents with perinatal HIV infection, receiving cART. | Design:  Cross-sectional study  Methods:  Questionnaires:   - PedsQL-self report - PedsQL-proxy report - CBCL | N=27  Age: 5-18 years  Perinatally infected  Treated with cART, only 19 out of 27 had complete viral suppression (<50 copies/ml).  Of the control group, all lived with biological parents, compared to 63% of HIV-infected children (p<0.05). | N=27  Randomly selected from local-area schools, matched by age and gender | General school functioning:   - The HIV+ group scored significantly lower on school functioning in the PedsQL-self report and PedsQL-proxy report compared to the control group (median 57.5 HIV+ vs 80 control, p= 0.000) resp. median 65 HIV+ vs 80 control p=0.004). - The HIV+ group scored significantly lower on the school competence scale of the CBCL compared to the control group (median 4.0 HIV+ vs 5.0 control p =0.004). |
| Brackis-Cott, E., et al.  2009a  USA | To describe the language ability and school functioning of early adolescents with perinatal HIV/AIDS. | Design:  Cross-sectional study  Methods:   - Interviews with HIV+ adolescents and their caregivers - Questionnaires including the Supervision and Involvement Scale of the Pittsburg Youth Study and Monitoring the Future survey - Reading subtest of the WRATIII. | N=43  Age: 9-15 years  Perinatally infected  Medical treatment unclear. | Populations norms | Need for special educational services and special education:   - 21% of the children with HIV attended special education. - 12% of the children with HIV attended a resource room or transitional class. - 49% of the children with HIV ever attended special education class. - Reasons for special education class were reading problems (57%), math problems (38%), attention problems (33%) and discipline problems (33%).   Repeating classes:   - 35% of the children with HIV repeated a class.   School attendance and drop-out:   - 23% of the children with HIV was ever suspended from school.   Reading, spelling and mathematics:   - Reading: 40% of the children with HIV scored below average (<25^th^ percentile) and 24% (<10^th^ percentile) on reading ability. - Reading: The mean WRAT3 score was 91.72 (SD=18.25) vs population norms 100 (SD=15). |
| Brackis-Cott, E., et al.  2009b  USA | To describe and compare the receptive language ability, word recognition skills and school functioning of older school-aged children and adolescents perinatally infected. | Design:  Cross-sectional study  Methods:   - Interviews with caregivers and children - Reading Subtest of the WRAT III | N= 206  Age: 9-16 years  Perinatally infected  84% cART  Fewer HIV-infected children were living with a biological parent (thus HIV+ caregiver) than the HEU children (p<0.05). | N=134  Age: 9-16 years  HEU children  Population norms | Need for special educational services and special education:   - 37% of the HIV+ group and 28% of the control group attended special education classes. - 52% of the HIV+ group and 40% of the control group had a history of special education placement, which is a significant difference.   Repeating classes:   - 37% of the HIV+ group and 31% of the control group repeated a class.   School attendance and drop-out:   - 99% of the HIV+ vs. 100% of the control group was currently in school.   Reading, spelling and mathematics:   - 54% of the HIV+ children and 40% of the HEU children scored below average (<25^th^ percentile) and 33% vs. 21% scored <10^th^ percentile on reading ability. - The HIV+ children scored significantly lower (mean 88.23, SD=17.92) on reading ability compared to the control group (mean 93.8, SD=17.72), p=0.008. - Reasons for special education placements for both HIV+ and the control group were mostly reading problems and math problems. |
| Chiriboga, C.,  et al.  2005  USA | To describe neurological outcomes in HIV-infected children in the cART-era including rates of progressive HIV encephalopathy (PHE) and clinical sequelae among PHE survivors | Design:  Cohort study  Methods:   - Neurological examinations, including queries regarding child behavior and scholastic performance | N= 126  Age: 0- ?  (n=96 in school-age)  Perinatally infected  60% cART  9% no medication | - | Need for special educational services and special education:   - 28% of the school-aged children were in special education classes. |
| Cohen, S. et al.  2015  The Netherlands | To compare the health-related quality of life of HIV+ children to healthy, SES-matched controls and the Dutch norm population. | Design:  Cross-sectional study  Methods:  - Questionnaires for parents/caregivers regarding sociodemographic data.  - Historical HIV viral load, CD4 counts, CDC classification from database Dutch HIV monitoring foundation  - PedsQL self-report, version 8-12 years and 13-18 years. | N=33  Age: 12-15 years  Perinatally infected  90% cART  6% on cART previously  3% had never been on cART  More HIV-infected children were born outside the Netherlands (p<0.001).  None of the healthy controls were adopted (p<0.001). | N=37  Age: 11-15 years  Healthy controls matched on age, gender, ethnicity and SES  &  National norms | Need for special educational services and special education:   - 21% of the HIV-infected children compared to 3% of the healthy controls were in special education school (primary or high), p-value not shown.   General school functioning   - On the PedsQL 8-12 years version (n=14 HIV+, n=22 control group), the HIV+ group had a mean score of 74.6 (SD:11.5) compared to the control group with mean score 76.6 (SD:14.5) and 78.9 (SD:11.9) for the Dutch norm population.   HIV vs control group p=0.674.  HIV vs Dutch norm p=0.199.   - On the PedsQL 13-18 years version (n=19 HIV+, n=15 control group), the HIV+ group scored 74.2 (SD:14.3) compared to the control group with 75.3 (SD:15.5) and 76.0 (SD:12.7) for the Dutch norm population.   HIV vs control group p=0.828.  HIV vs Dutch norm p=0.581.   - The proportion of HIV-infected children with an impaired school subscale score (≥1SD below the mean of the norm population) was 27% in the HIV+ group, 22% in the control group and 14% in the Dutch norm population. HIV vs Dutch norm p=0.045. |
| Dolfus, C., et al.  2010  France | To describe the living conditions and clinical and immunovirological outcomes of HIV1-infected children born before December 1993. | Design:  Cohort study  Methods:  - Questionnaires for HIV+ children and their mothers, at 6 month intervals | N=179  Age: born between 1985-1993, median 15 years  Perinatally infected  77.1% cART  4.8 % Dual therapy  18.1% no treatment | General population (same age group) | Need for special educational services and special education:   - 2.7% of the children with HIV required special education.   General school functioning:   - 67.0% of the adolescents was in general schooling with academic success. - 17.3% of the adolescents was in general schooling with academic failure; repeating 2 or more grades (15%) or dropped out of school (2.2%). - Academic failure is similar to that for this age group in the general population (16.5%). |
| Ellis, W.  2004  USA | To identify HIV-related medical, academically-based and affective/social factors associated with the academic achievement of perinatally HIV-infected children | Design:  Cross-sectional study  Methods:  - Self-designed questionnaires for mothers of HIV-infected children  - End-of-Grade test in reading and mathematics to measure academic achievement | N=9  Age: in elementary and middle school  Perinatally infected  Medical treatment unclear | Children from the same rural county | School attendance and drop-outs:   - 44% of the mothers cited truancy as problem of their child.   Reading, spelling and mathematics:   - Reading: 56% failed proficiency levels in reading in the End-of-Grade tests, compared to 26% in children from the same rural county. - Mathematics: 56% failed proficiency levels in mathematics in the End-of-Grade tests, compared to 8% in children from the same rural county.   Social participation at school:   - Affective/social problems and behavioural problems were reported frequently in the school setting, containing low self-esteem: 89%, peer pressure: 78% and disruptive behavior 78%. |
| Ellis, W.  2010  USA | To describe the extent to which HIV-infected and HIV-negative children were referred to social work and counselling services because of episodes of behavioural crisis in school and underlying precipitators of those crises. | Design:  Cross-sectional study  Methods:  - Self-designed questionnaires with questions regarding children’s behavioural crises in school during the past academic school year. | N=37  Age: school-age (mean age: 10 years)  Perinatally infected  Medical treatment unclear | N=95  Age: school-age (mean age: 13 years)  HEU children | Social participation at school:   - 41% of the HIV-infected mothers reported that one or more of their children exhibited a behavioural crisis in school. - Sexual harassment of classmates and being verbally abusive to classmates or teachers were the most frequently occurring externalizing problems for HIV-positive children. |
| Ellis, W.  2011  USA | To investigate the relationship between depression in HIV-infected mother and their children’s behavioral crises and to describe precipitators of these crises. | Design:  Cross-sectional study  Methods:  - Self-designed questionnaire with questions regarding children’s behavioural crises in school during the past academic school year.  - Beck Depression Inventory (BDI) | N=37  Age: school-age (mean age: 10 years)  Perinatally infected  Medical treatment unclear | N=95  Age: school-age (mean age: 13 years)  HEU children | Social participation at school:   - Disruptive behaviour and disciplinary issues were the main externalizing problems for HIV-positive children of depressed mothers. |
| Franklin, S., et al.  2007    USA | To investigate the behavioral adjustment and academic status of children with HIV infection through serial evaluations over a ten-year period. | Design:  Cohort study  Methods:  - Tests: Woodcock Johnson Tests of Achievement-R and III | N= 25  Age: 4-12 years  Perinatally infected  Cohort started before cART was available, since 1996, 64% has used cART. | National norms | Reading, spelling and mathematics:   - Reading:   Mean scores were 88 at age 6 to 94 at age 12 compared to the national norm with mean score 100 (SD:15).   - Spelling:   Mean scores were 89 at age 6 to 74 at age 13 compared to the national norm with mean score 100 (SD:15).   - Mathematics:   Mean scores were 81 at age 4 to 88 at age 13 compared to the national norm with mean score 100 (SD:15). |
| Fundaro, C., et al.  1998  Italy | To explore the differences in neuropsychologic development of HIV positive children compared with HEU children. | Design:  Cross-sectional study  Methods:  -The Cornoldi MT reading tests  - The Cornoldi PRCR2 prerequisite test for diagnosing reading and writing difficulties  - Writing test standardized by age | N=8  Age: 6-12 years  Perinatally infected  75% cART  25% no medication  Asymptomatic during the study, all were exposed to heroin or methadone during fetal life and were appropriate for gestational age and birth weight. | N=8  Age: 5.6-11.7 years  HEU children  Comparable in terms of prenatal drug exposure, SES and cultural environment. | Need for special educational services and special education:   - 75% of the HIV-positive children have a special support teacher.   Reading, spelling and mathematics:   - Reading: Reading tests showed to be poor on average in 7/8 children and good in 1/8 for HIV-positive children. - Reading: HIV-positive children had some degree of comprehension difficulties in the reading tests, whereas HEU children performed in line with their school age. - Spelling: 60% of the HIV-positive children showed spelling mistakes.   General school functioning:   - All of the HIV-positive children had poor school achievement, as confirmed by their school report. |
| Gadow, K., et al.  2010  USA | To compare the rates of psychopathology in HIV+ youths with a sample of peers. | Design:  Cross-sectional study  Methods:  - Questionnaires for parents for demographics  - School Functioning subscale of the Social and Academic Functioning Questionnaire (high value indicates poor functioning) | N=319  Age: 6-17 years  Perinatally infected  81% cART  Of the control group, more children had a biological parent as primary caregiver (p<0.001) and came from lower income households (p<0.001). | N= 256  Age: 6-17 years  HEU children (68%) or living in a household with HIV-infected family members (32%). | Need for special educational services and special education:   - Of the HIV+ group 44% was ever in special education, compared to 32% of the control group (p=0.004).   General school functioning:   - The HIV+ group scored significantly higher (poorer) on academic functioning (mean 2.7, SD 2.2) compared with the control group of peers (mean 2.0, SD 2.1) (p<0.001). |
| Garvie, A., et al.  2014  USA and Puerto Rico | To evaluate academic achievement in youth with HIV and HIV-exposed uninfected peers. | Design:  Cross-sectional study  Methods:   - Wechsler Individual Achievement Test,2^nd^ edition (WIAT-II-A) | N=295  Age: 7-16 years  Perinatally infected  90% cART  More HEU children  had a biological parent as primary caregiver (p<0.001) and came from lower income households (p<0.001). | N=167  Age: 7-16 years  HEU children  National norms | Reading, Spelling and Mathematics;  Reading:   - Children with HIV did not differ on Word Reading scores with HEU children (mean 87.7 vs. 90.5, p=0.07). - Children with HIV and HEU children scored significantly lower on Word Reading compared to national norm scores (mean 100), p<0.001).   Spelling:   - Children with HIV did not differ on Spelling scores with HEU children (mean 90.5 vs. 91.8, p=0.42). - Children with HIV and HEU children scored significantly lower on Spelling compared to national norm scores (mean 100), p<0.001.   Mathematics:   - Children with HIV scored significantly lower on Numerical Operations compared with HEU children (mean 82.0 vs 88.8, p<0.001). - Children with HIV and HEU children scored significantly lower on Numerical Operations compared to national norm scores (mean 100), p<0.001.   General school functioning:   - Children with HIV scored significantly lower on total academic achievement compared with HEU children (mean 86.0 vs 89.2, p=0.04). - Children with HIV and HEU children scored significantly lower on total academic achievement (TA) compared to national norm scores (mean 100), p<0.001. |
| González-Tomé, M., et al.  2018  Spain | To assess sleep quality in a cohort of HIV-infected adolescents and determine the impact of antiretroviral therapy on sleep. | Design:  Cross-sectional study  Methods:   - Interviews with patients regarding their school performance | N=46  Age: median age 16 years  Perinatally infected  100% cART  23.9% showed some event linked to AIDS category at the time of enrollment. |  | Repeating classes:   - 30.4% of the children with HIV repeated 1 grade. - 30.4% of the children with HIV repeated 2 or more grades.   General school functioning:   - 43.5% of the children with HIV classified their school performance as poor. - 13.0% of the children with HIV classified their school performance as ‘with difficulties’. |
| Jeremy, R., et al.  2005  USA and Puerto Rico | To investigate neuropsychological functioning and its correlation with viral load for previously treated HIV+ children who underwent a change in treatment regimen. | Design:  Prospective cohort  Methods:  - Learning scale of Conners’ Parent Rating Scales (CPRS) | N=420  Age: 3-17 years  Perinatally infected  100% cART 16 weeks before study entry, during study randomized onto 1 of 7 drug treatment combinations. | National norms | Learning:   - The HIV+ group scored significantly higher on learning problems at baseline (mean 57.6, SD: 16.6) compared to national norms (mean 50.0, SD:10) p<0.001. |
| Kullgren, K., et al.  2004  USA | To describe and predict cognitive, adaptive and behavioral  functioning in HIV+ children utilizing a risk factor model. | Design:  Cross-sectional study  Methods:  - Learning scale of Conners’ Parent Rating Scales (CPRS) | N=67  Age: 3-16 years  Perinatally infected  93% antiretroviral treatment  18% were born premature. 58% had been prenatally drug exposed. 31% AIDS diagnosis at the time of their evaluation. | National norms | Learning:   - The HIV+ group scored significantly higher on learning problems (59.02, SD:17.29) compared to national norms (50.0, SD:10) p<0.05. - 25% of the children had scores on the learning scale that fell in the clinical range (>2SD below mean). |
| Lichtenstein, B.  2010  USA | To examine psychosocial stressors for family members who are affected by HIV/AIDS | Design:  Qualitative study  Methods:   - Interviews with social workers - Chart review of case histories of clients with HIV/AIDS | N=141 clinical charts (49 children/ adolescents with HIV)  N=12 social workers  Age: < 2 years  Transmission unclear (most likely perinatally infected because their mothers also have HIV)  Medical treatment unclear | - | Learning:   - A lot a children have learning difficulties.   Social participation at school:   - The children were taunted at school and got into a lot of fights at school. |
| Malee, K., et al.  2011  USA and Puerto Rico | To examine the behavioral functioning of HIV+ youth and to evaluate the relationship between behavioral functioning and medication adherence. | Design:  Cross-sectional analysis of data from a prospective cohort study  Methods:  - Learning scale of Conners’ Parent Rating Scales (CPRS)  - Caregiver reports of events occurred or not for participants over 5 years: repeating a grade, receiving special help in school, having limitations in physical activity or school attendance | N=1134  Age: 3-17 years  Perinatally infected  100% ART  25% had CDC class C classification, 15% had a neurologic diagnosis, 17% had a psychiatric diagnosis. | National norms | Need for special educational services and special education:   - 36 % of the parents reported that their HIV+ child attended a special class/help at school.   Repeating classes:   - 24% of the parents reported their HIV+ child had repeated a grade in the past 5 years.   School attendance and drop-outs:   - 13% of the parents reported limited school attendance of their HIV+ child in the past 5 years.   Learning:   - The HIV+ group scored significantly higher on learning problems (mean 55, SD:15) compared to national norms (mean 50.0, SD: 10) p<0.001.   The above SDs were rounded to the nearest integer.   - Higher than expected rates of the children demonstrated impairment in learning: 22% (T>65), p<0.0001. |
| Medin, G., et al.  2016  Spain | To assess the psychosocial, emotional and behavioral functioning in a cohort of HIV+ adolescents. | Design:  Cross-sectional study  Methods:  - Caregiver reports of events occurred or not for participants over 5 years: repeating a grade, receiving special help in school, having limitations in physical activity or school attendance | N=95  Age: 11-19 years  Perinatally infected  92.6% cART  34% currently AIDS category, 18% HIV-encephalopathy. The mode of transmission by the mothers was mainly due to drug abuse. | - | Need for special educational services and special education:   - 53.6% of the children with HIV needed extra support at school.   Repeating classes:   - Nearly of the children with HIV 40% had been held back one school year. - 20% of the children with HIV had been held back two or more years.   School attendance and drop-outs:   - 90.1% of the children with HIV attended school.     Social participation at school:   - 17.4% of the children disclosed the HIV-status at school. |
| Mellins,C, et al.  2003  USA and  Puerto Rico | To examine the influences of HIV infection, drug exposure and family characteristics on behavioral outcomes in children | Design:  Cohort study  Methods:  Every 6 months, beginning at age 3  - Learning scale of Conners’ Parent Rating Scales (CPRS)  (average of 4.1 sets of CPRS scores were collected for each child)  - Demographic data were collected from the primary caregiver each study visit | N= 96 HIV+  Age: 3-17 years  Perinatally infected  27% AIDS during the course of the study.  Children with HIV were more likely than control group to have been born prematurely (p=0.0006), had a low birth weight (p=0.03), had in utero exposure to heroin (p=0.03) and in care of somebody other than their birth mother (p=0.01).  Medical treatment unclear | N=211  Age: 3-17 years  HEU children | Learning:   - The HIV+ children did have similar scores on learning problems compared to the control group (p=0.710). |
| Mialky, E., et al.  2001  USA | To explore medical, educational and psychosocial aspects of HIV+ children | Design:  Cross-sectional study  Methods:  - Surveys for patients and their caregivers  - Retrospective review of medical records | N=85 (N=54 surveys regarding school-related topics)  Age: 5-18 years  Perinatally infected  50.6% had severe symptoms related to HIV-infection (CDC category C)  Medical treatment unclear | - | Need for special educational services and special education:   - 52% of the children with HIV received some special educational services in their school. - 13% of the children with HIV were in full-time special education classes.   Repeating classes:   - 23.5% of the children with HIV repeated at least one grade.   School attendance and drop-outs:   - The children with HIV missed an average of 5.4 days in the last quarter term.   Social participation at school:   - 23% had told a school official of their child’s HIV infection. - School nurses were the most frequently informed school employees about the HIV-status. |
| Nachman, S., et al.  2012  USA and Puerto Rico | To evaluate associations between HIV disease severity and psychiatric  and functional outcomes in youth with perinatal  HIV infection. | Design:  Cross-sectional analysis of data from a prospective cohort study  Methods:  - School Functioning subscale of the Social and Academic Functioning Questionnaire (high value indicates poor functioning)  - Treatment data with current or past exposure to efavirenz  - DSM-IV referenced rating scales, The Child and Adolescent Symptom Inventory-4R (CASI-4R) | N=319  Age: 6-17 years  Perinatally infected  89% cART  3% other combination therapy  8% no ARVs | - | General school functioning:     - Educational problems are common in youth with perinatal HIV-infection. |
| Nozyce, M., et al.  2006  USA | To characterize behavioral and cognitive profiles of clinically and immunologically stable HIV+ children. | Design:  Cross-sectional study  Methods:  - Learning scale of Conners’ Parent Rating Scales (CPRS)  - Wechsler Preschool and Primary Scales of Intelligence-Revised  - Wechsler Intelligence Scale for Children-3^rd^ edition (WISC-III) | N=274  Age: 2-17 years  Transmission route not described.  Monotherapy (31%), other monotherapy (13%), combination of zidovudine and didanosine (44%) or other combination therapy (13%)  All children had stable CD4 cell number or percentage maintained in Centers for Disease Control and Prevention immune category 1 or 2 during the 4 months before study entry. | National norms | Learning:   - 25% of the children were classified as having a learning problem (scored>65 = >1.5 SD). |
| Rehm, R. & Franck, L.  2000  USA | To examine strategies used by families to manage the consequences  of children's HIV disease. | Design:  Qualitative study  Methods:  - Interviews with children, parents and other caregivers | N=21 (N=9 children)  Age: 7-15 years  Respondents from 8 different families.  89% perinatally infected  11% infected through blood transfusion  100% medication, 78% with protease inhibitors. | - | Social participation at school:   - Stigma and disclosure at school are important issues for children with HIV and their parents. - Selective disclosure has been used as a strategy to reduce stigma. - Another strategy to reduce stigma was controlling children’s social lives outside of the family to minimize the need for disclosing; reducing overnight stays at classmates or make sure children can be responsible for their medication when away from home. |
| Sirois, P., et al.  2016a  USA and Puerto Rico | To examine the relationship between the use of commonly prescribed stimulants and changes in measures of cognition, behavior and QOL in HIV+ children. | Design:  Cohort study  Methods:  - Learning scale of Conners’ Parent Rating Scales (CPRS)  - National Health Interview Survey, social/school functioning subdomain | N= 524  Age: 3-16 years  Perinatally infected  69.6% HAART with PI, 7.6% HAART without PI, 18.7% other ARV regimen,  4.0% no ART | Comparison within the group of HIV+ children: n=132 with prescriptions for stimulants (PG), n=392 matched controls (CG)  National norms | Learning:   - Children with HIV scored significantly higher (more learning problems) compared with national norms. The PG scored mean 63.0, SD:16.9, compared with the CG mean 52.8, SD: 15.4 and national norms mean 50.0, SD: 15.0. |
| Sirois, P., et al.  2016b  USA | To examine the association of memory and executive function with academic and adaptive skills among youth with PHIV and HEU children. | Design:  Cross-sectional study  Methods:  - Wechsler Individual Achievement Test,2^nd^ edition (WIAT-II-A) | N= 173  Age: 9-18 years  Perinatally infected  93% of the PHIV without CDC class C diagnosis were prescribed ARVs and 95.6% of PHIV with CDC class C diagnosis.  More HEU children had a biological parent as primary caregiver (p<0.001) and came from lower income households (p=0.002).  9.2% of the HIV-infected children had been diagnosed with encephalopathy prior to or at study entry. | N= 85  Age: 9-18 years  HEU children  National norms  Comparison within the group of PHIV , with and without CDC class C diagnosis. | Reading:   - Children with HIV had average (PHIV/non-C, mean score 92.1) to low average Reading scores (PHIV/C, mean score 82.6), compared to national norm scores (mean: 100). - Children with HIV had higher-than-expected rates of impairment in reading (2 SDs below the mean) compared to national norms. - Children with PHIV/C had significantly lower (worse) reading scores compared with HEU children.   Mathematics:   - Children with HIV had low-average (PHIV/non-C, mean score 85.1) to borderline scores (PHIV/C, mean score 77.7) on Numerical Operations compared to national norm scores (mean: 100). - Children with HIV had higher-than-expected rates of impairment in mathematics (2SDs below the mean) compared to national norms. - No significant differences were found between children with PHIV/C, PHIV/non-C and HEU children. |
| Storm, D. et al.  2005  USA  and Puerto Rico | To examine quality of life (QOL) among HIV+ children and compare QOL outcomes between treatment groups that differ in medication. | Design:  Cross-sectional study  Methods:  - National Health Interview Survey, social/school functioning subdomain | N=940  Age: 5-18 years  Perinatally infected  72% PI therapy  28% Non-PI therapy  14% of the children had severe immune suppression. 28% met criteria for AIDS at study entry. | Comparison within the total group; n=677 with PI therapy, n=263 with Non-PI therapy. | Need for special educational services and special education:   - 32% of the children with HIV receives special help in school.   Repeating classes   - 21% of the children with HIV reported repeating a grade.   School attendance and drop-outs   - 17% of the children with HIV reported limitations in school attendance.   General school functioning:   - For 58% of the children, one or more limitations in social/school functioning were reported. |
| Vuppula, S., et al.  2017  USA | To review clinical, laboratory and social outcomes of perinatally HIV-infected children born in the pre-ART, ART and HAART era. | Design:  Retrospective, cross-sectional study  Methods:   - Abstraction from clinical and laboratory database. | N=69  Age : <19 years (with retrospective data on educational achievement level)  Perinatally infected  Medical treatment of subgroup unclear. | Children from the same city. | School attendance and drop-outs:   - 81% of the cohort old enough to finish high school has graduated compared with an average of 66.2% graduation rate of students from New York City during the same time period, which is a significant difference (p-value not shown). |
| Wolf, E., et al.  2016  USA | To investigate the short-term and medium-term health outcomes of HIV-infected foreign-born adoptees. | Design:  Retrospective cohort study  Methods:   - Abstraction from medical records | N= 79  Age: 0-20 years  Adoptees.  Transmission route not described.  75% on ART.  Of the children on ART, 76% had suppressed viral load at baseline. |  | Need for special educational services and special education:   - Few children had individualized education programs, tutoring, special education classes and early-intervention programs. - 45% of the children were home-schooled.   Repeating classes:   - Many of the children were below expected grade level for age in school (40% at follow-up). |
| Wood, S., et al.  2009  USA | To explore the association between previous severe HIV disease, defined as past CDC class C diagnosis and neurocognitive and psychiatric outcomes in long-term survivors with HIV. | Design:  Retrospective cohort study  Methods:  Review of medical record, searching for:  - Learning disability (defined as a discrete impairment in at least one area of learning, with diagnosis confirmed with psychometric testing).  - Educational learning support (defined as having a formal individualized educational plan through the school system or attending specialized classes). | N=81  Age: 11-23 years  Perinatally infected  93% cART at end of study period.  47% class C diagnosis  53% no class C diagnosis | Comparison within the total group, n=38 class C diagnosis, n=43 No class C diagnosis. | Need for special educational services and special education:   - 35.8% were receiving specialized learning support in school.   Learning:   - 42% of the total group had been diagnosed with a learning disability. |
