# Supplementary material for: School functioning of children with perinatal HIV-infection in high-income countries: A systematic review
Source: PLoS One. 2021 Jun 4;16(6):e0252746. doi: 10.1371/journal.pone.0252746 (PMC8177442; doi:10.1371/journal.pone.0252746)
Supplement: S4 Appendix — (DOCX) [file pone.0252746.s005.docx]

# S4 Appendix. General school functioning scores

|  | **Measure of school functioning** | **N HIV+** | **N control** | **Type of control** | **Difference** |
| --- | --- | --- | --- | --- | --- |
| Bomba et al., 2010 | - Child Behavior Checklist - Pediatric Quality of Life Inventory-self report - Pediatric Quality of Life Inventory-proxy report | 27 | 27 | - Healthy controls matched by age and gender | p=0.004  p=0.000  p=0.004 |
| Cohen et al., 2015 | - Pediatric Quality of Life Inventory-self report | 33 | 37 | - Healthy controls matched on age, gender, ethnicity and SES - National norm scores | p>0.05 (exact data not shown)  p=0.045 |
| Gadow et al., 2010 | - Social and Academic Functioning Questionnaire | 319 | 256 | - HEU children or children living with HIV+ family members | p<0.001 |
| Garvie 2014 | - Wechsler Individual Achievement Test | 295 | 167 | - HEU children - National norm scores | p=0.04  p<0.001 |

*General school functioning scores of children with HIV compared to national norm scores, matched healthy controls and HEU children/children living with HIV+ family members.*
